# Acupuncture Enhances Communication between Cortices with Damaged White Matters in Poststroke Motor Impairment

**DOI:** 10.1155/2019/4245753

**Published:** 2019-01-02

**Authors:** Xiao Han, Lijun Bai, Chuanzhu Sun, Xuan Niu, Yanzhe Ning, Zhen Chen, Yingying Li, Kuangshi Li, Diyang Lyu, Caihong Fu, Fangyuan Cui, Zhengguang Chen, Zhongjian Tan, Lixin Tang, Yihuai Zou

**Affiliations:** ^1^Department of Neurology and Stroke Center, Dongzhimen Hospital, The First Affiliated Hospital of Beijing University of Chinese Medicine, Beijing, China; ^2^The Key Laboratory of Biomedical Information Engineering, Ministry of Education, Department of Biomedical Engineering, School of Life Science and Technology, Xi'an Jiaotong University, Xi'an, China; ^3^Department of Medical Imaging, The First Affiliated Hospital of Xi'an Jiaotong University, Xi'an, China; ^4^The National Clinical Research Center for Mental Disorders & Beijing Key Laboratory of Mental Disorders, Beijing Anding Hospital, Capital Medical University, Beijing, China; ^5^Department of Internal Medicine, Gulou Hospital of Traditional Chinese Medicine of Beijing, Beijing, China; ^6^Department of Rehabilitation, Shunyi Hospital Affiliated to Beijing Hospital of Traditional Chinese Medicine, Beijing, China; ^7^Department of Radiology, Dongzhimen Hospital, The First Affiliated Hospital of Beijing University of Chinese Medicine, Beijing, China; ^8^Department of Acupuncture, Dongzhimen Hospital, The First Affiliated Hospital of Beijing University of Chinese Medicine, Beijing, China

## Abstract

Stroke is a leading cause of motor disability. Acupuncture is an effective therapeutic strategy for poststroke motor impairment. However, its mechanism is still elusive. Twenty-two stroke patients having a right-hemispheric subcortical infarct and 22 matched healthy controls were recruited to undergo diffusion tensor imaging (DTI) and functional magnetic resonance imaging (fMRI) scanning. The resting-state fMRI was implemented before and after needling at GB34 (Yanglingquan). The stroke patients presented a substantially reduced fractional anisotropy value in the right superior longitudinal fasciculus (SLF), corticospinal tract, and corpus callosum. The structural integrity of the frontoparietal part of the SLF (SLF-FP) correlated with the motor scores of lower limbs in stroke patients. This corticocortical association bundle originated from the premotor cortex (PM) and the adjacent supplementary motor area (SMA), known as secondary motor areas, and terminated in the supramarginal gyrus (SMG). After acupuncture intervention, the corresponding functional connectivity between the PM/SMA and SMG was enhanced in stroke patients compared with healthy controls. These findings suggested that the integrity of the SLF is a potential neuroimaging biomarker for motor disability of lower limbs following a stroke. Acupuncture could increase the communication between the cortices connected by the impaired white matter tracts, implying the neural mechanism underlying the acupuncture intervention.

## 1. Introduction

Stroke is a critical contributor to the risk of death and a common cause of acquired disability on a global scale [1–3]. The most notable outcome of stroke is motor impairment. A number of functional magnetic resonance imaging (fMRI) studies have engaged in mapping the functional alterations following a stroke and proposed that motor impairment is relevant to abnormal functional patterns [4, 5]. Meanwhile, the structure damage also played a key role in motor disability [6, 7], especially the location of the lesion [8]. After an ischemic stroke, disruptive changes in structure and disorganized patterns in function are independently associated with the impaired mobility and also have the reciprocal correlation [9].

Abundant emerging studies [10–12] have focused on mapping the relationship among structural damage, functional alterations, and behavioral deficits of motor loss following the stroke using diffusion tensor imaging (DTI) and fMRI. DTI can detect the diffusion direction of water molecules along the course of the axonal tract to delineate white matter architecture by extracting the fractional anisotropy (FA) value [13, 14]. Tract-based spatial statistics (TBSS) analysis with DTI allows for the quantitative measure of the microstructure disruptions in white matter tracts following a stroke [15–17]. The functional connectivity (FC) is a statistical temporal dependence between neurophysiological recordings acquired from spatially distinct brain regions [18]. The regions that have a high degree of temporal correlations are presumed to share functional properties [19]. It is imperative to probe functional alterations based on structural disruptions coupled with motor deficits so as to elucidate the pathophysiological features of impaired mobility after the stroke.

Acupuncture, as a widely recognized therapeutic strategy, has been used for more than 2000 years [20]. Increasing evidence from empirical and clinical studies suggests that acupuncture is effective in ameliorating motor impairment after brain lesions in a stroke [21–25]. Acupuncture at acupoint GB34 could enhance FC between the bilateral primary motor cortices, which decreased due to the stroke [22]. At the same time, it was observed using DTI that acupuncture therapy could protect white matter integrity in stroke patients by postponing Wallerian degeneration [26]. Nevertheless, with the structurally damaged white matters, it is elusive whether acupuncture could make an effect on communication between regions linked by injured tracts. The cortical gray matters with coupled functional activation are expected to have communication in white matters [12, 27]. Accordingly, studies are needed to combine the severity of motor deficits with disruptive structural white matter and FC patterns so as to further understand the mechanisms of acupuncture therapy for motor disability in stroke patients [27]. The present study examined the stroke-induced structural damage and the following functional abnormalities associated with motor impairment. It was hypothesized that acupuncture could modulate these functional alterations despite the disruption of the corresponding structural integrity.

## 2. Materials and Methods

### 2.1. Participants

Twenty-two ischemic stroke patients (15 male, aged 59.9 ± 7.7 years) having right-hemispheric subcortical infarcts were recruited. None had a history of neurological or psychiatric disorders. Conventional magnetic resonance images (MRI) did not find any abnormalities except for the infarct lesion in each patient. The inclusion criteria were as follows: first-ever ischemic stroke; single ischemic lesion restricted to the right internal capsule, basal ganglia, corona radiata, and its neighboring regions; within 3 months from stroke onset to the MRI scan; aged 35–75 years; and without psychiatric disorders. The exclusion criteria were as follows: any brain abnormalities except infarction; any other physical or psychiatric conditions that may influence participation; and any MRI contraindications. Twenty-two healthy controls (10 male, aged 58.0 ± 5.2 years) were enrolled with no history of neurological or psychiatric disorders. All of the participants were right-hand dominant with right-hemispheric lesions to control the dominant effect of the left hemisphere. The purpose and procedure of the study were explained in detail to each participant, including the imaging session and the acupuncture intervention. We confirmed with the participants that they were able to stay about 30 mins in the fMRI scan. All participants were informed that they could terminate the experiment at any time. The signed consent was obtained from all participants. This study got ethical approval from the Ethics Committee of Beijing University of Chinese Medicine and was conducted in accordance with the Declaration of Helsinki.

### 2.2. Clinical Assessments

A series of neurological examinations were performed, including the National Institute of Health Stroke Scale (NIHSS) for stroke-related neurologic deficits [28] and the Fugl-Meyer Assessment (FMA) for a quantitative measure of motor disability [29, 30]. All patients underwent these clinical assessments (shown in Table 1).

### 2.3. Imaging Acquisition Protocol

Images were acquired on a 3.0-Tesla MRI scanner (Siemens, Germany) at Dongzhimen Hospital, Beijing, China. All participants were asked to rest for 20 min and researchers would confirm again that the participants were completely understood the experiment procedure. Subsequently, they were instructed to stay still, think of nothing, in particular, keep eyes closed, and not to fall asleep during scanning. Earplugs were worn to attenuate scanner noise, and foam head holders were immobilized to minimize head movements during scanning. If the sensation was considered as uncomfortable, the participants could terminate the imaging session at any time without consequences.

Prior to the functional scanning, high-resolution structural information for anatomical localization was acquired using 3D MRI sequences. A single-shot, gradient-recalled echo-planar imaging sequence was applied to collect the resting-state fMRI data with the following parameters: repetition time, 2000 ms; echo time, 30 ms; flip angle, 90°; matrix, 64 × 64; field of view, 225 × 225 mm^2^; slice thickness, 3.5 mm; gap, 1 mm, 32 interleaved axial slices; and 180 volumes.

The DTI data were obtained with a single-shot, gradient-recalled echo-planar imaging sequence. The diffusion sensitizing gradients were applied along 30 noncollinear directions (*b* = 1000 s/mm^2^) with an acquisition without diffusion weighting (*b* = 0 s/mm^2^). The imaging parameters were as follows: repetition time, 18,000 ms; echo time, 94 ms; flip angle, 90°; matrix, 160 × 160; field of view, 256 × 256 mm^2^; slice thickness, 1.5 mm; and 80 contiguous axial slices.

Subsequently, the acupuncture intervention and a consequent fMRI scan with the same parameters of resting-state scan were applied. Finally, structural imaging was performed using a T1-weighted 3D anatomical sequence. Supplementary Figure 1 shows the experiment design.

### 2.4. Acupuncture Intervention

The nonrepeated event-related fMRI (NRER-fMRI) design [31] used in a previous study was adopted [22]. In brief, following a resting-state fMRI scan and a DTI scan, the participants underwent acupuncture intervention and another fMRI scan without removing the needle but no manipulation. The acupuncture intervention included 1-min even reinforcing and reducing manipulation (rotating the needle clockwise and counterclockwise at 1 Hz for 60 s). Before the manipulation, a sterile, single-use silver needle (40 mm in length and 0.30 mm in diameter) was vertically inserted at GB34 (Yanglingquan) to a depth of 2–3 cm. The acupoint GB34 was located in the lateral aspect of the posterior knee. GB34 is commonly used in clinical treatment for a stroke, especially motor impairment [32–34]. The same licensed and skilled acupuncturist performed the procedures. After each fMRI scanning, the degree of “deqi” sensation (experience during acupuncture, containing soreness, heaviness, fullness, or pressure) was recorded using a 10-point visual analog scale to ensure the proper process of acupuncture intervention [35]. The participants were excluded if they reported the sharp pain that was believed to be an inadvertent noxious stimulation. No one among the participants of the present study reported the sharp pain.

### 2.5. Multimodal fMRI Analysis

For DTI analysis, FA was generated using the TBSS in the FMRIB Software Library [36]. Image analysis using TBSS included the following steps: (1) nonlinear alignment of FA images of all participants into a common space using the FMRIB nonlinear registration tool; (2) affine transformation of the aligned images into the standard MNI152 1-mm space; (3) averaging of the aligned FA images to create a 4D mean FA image; (4) thinning of the mean FA image to create a mean FA “skeleton” that represented the centers of all white matter tracts common to the group; and (5) thresholding of the FA skeleton at FA 0.2 to suppress areas of extremely low mean FA and exclude those with considerable interindividual variability. Nonparametric, permutation-based tests were carried out in group comparison (stroke patients versus healthy controls) by Randomize [included in FMRIB Software Library (FSL)] with 5000 permutations and threshold-free cluster enhancement using age, sex, and poststroke duration as covariates. The threshold for statistical significance was set at* P* < 0.01, adjusted for multiple comparisons. Pearson correlation analyses were performed with a threshold cluster level of* P* < 0.05 (Gaussian Random Field, GRF corrected) to test the correlations between the structural imaging measures presented with the group difference and clinical variables.

Resting data preprocessing steps were carried out using the FSL (https://fsl.fmrib.ox.ac.uk/fsl/fslwiki). The first five volumes were discarded to eliminate T1 relaxation effects. The images were first slice time correction and then realigned to correct for head motions (none of the participants had head movements exceeding 1.5 mm on any axis and head rotation greater than 1 degree). The image data were further processed with spatial normalization based on the Montreal Neurological Institute (MNI) space and resampled at 3 × 3 × 3 mm^3^. The smoothing used Gaussian filters of 6-mm full width at half maximum, spurious variance (head motion, ventricular and white matter signal, and derivatives of each of these signals) reduction, and band-pass filtering (0.01–0.08 Hz).

The origins and terminations of the white matter trajectories which had significant correlations with the clinical variables were used as center coordinates of 10-mm-radius spherical regions of interest (ROIs). The cortical gray matter was extracted from the spherical ROIs using a cortical mask in the REST software (V1.8, http://restfmri.net/forum/index.php). The ROIs were selected for further FC analysis for the resting-state data collected pre- and postacupuncture intervention. Resting-state FC was performed using custom MATLAB scripts. The mean time course across all voxels within the ROI for each participant was extracted and used as the model response function in the general linear model. Temporal correlations were conducted among time series data of individuals between the aforementioned two ROIs. The correlation for FC was calculated with Fisher's Z-transformation using the REST software. Subsequently, analysis of variance (ANOVA) with repeated measures was performed on the FC values across the two groups (stroke patients versus healthy controls) and acupuncture condition (pre- and postacupuncture), where the former factor was between-participant and the latter within-participant. The main effects of group, acupuncture condition, and their interactions on FC values were examined. The two-sample* t* test was used to compare FC values between two groups during pre- and postacupuncture so as to investigate the effect of group on FC change, while the paired-samples* t* test was conducted by comparing pre- and postacupuncture intervention FC values for the stroke patients and healthy controls separately. In addition, the correlation between the FC values of pre- and postacupuncture intervention and the corresponding FA values was also checked using Pearson correlation analyses with* P* values less than 0.05 denoting statistical significance.

## 3. Results

### 3.1. Demographic and Clinical Features

Table 1 shows the basic demographic and clinical data. In this study, the subcortical ischemic lesions were located in the right hemisphere. The duration after the onset of stroke was restricted to 90 days (mean value, 41.68 ± 25.02 days). The results showed that all patients had motor hemiparesis with NIHSS from 1 to 14 (mean value, 5.05 ± 3.29), Fugl-Meyer Assessment of the upper limbs (FMA-U) from 6 to 65 (mean value, 34.27 ± 19.80), and Fugl-Meyer Assessment of the lower limbs (FMA-L) from 9 to 34 (mean value, 24.50 ± 8.62). No significant differences were found between stroke patients and healthy controls in terms of age (*P* = 0.328) and sex (*P* = 0.223).

### 3.2. Result from TBSS Analysis

**Figure 1:**
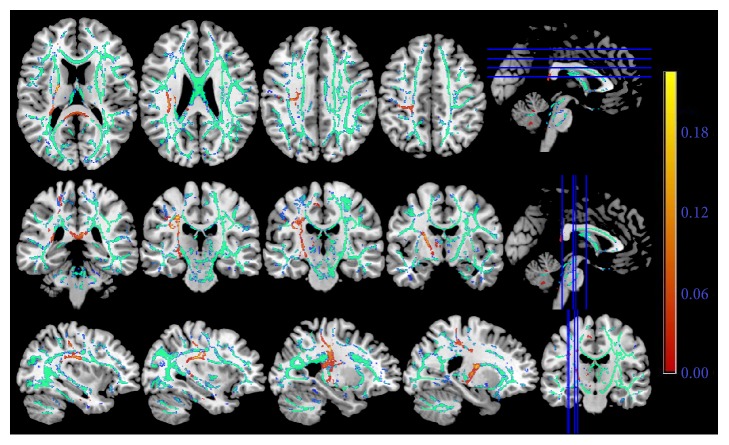
The FA skeleton based on the diffusion tensor imaging. The red color indicated the injured white matter fibers in the stroke patients in contrast to the healthy controls. The green color represents the common white matter tracts between two groups of participants. The right-hemispheric subcortical stroke patients displayed significantly decreased fractional anisotropy in the ipsilesional superior longitudinal fasciculus, corticospinal tract, and the corpus callosum.

The loss of structural integrity in certain white matter fibers in the patients with right-hemispheric subcortical infarct was figured out using TBSS. It illustrated that the right superior longitudinal fasciculus (SLF), corticospinal tract (CST), and the corpus callosum had significantly reduced FA value in stroke patients compared with healthy controls (*P *< 0.01, Figure 1).

### 3.3. Correlation between FA and Clinical Scores

**Figure 2:**
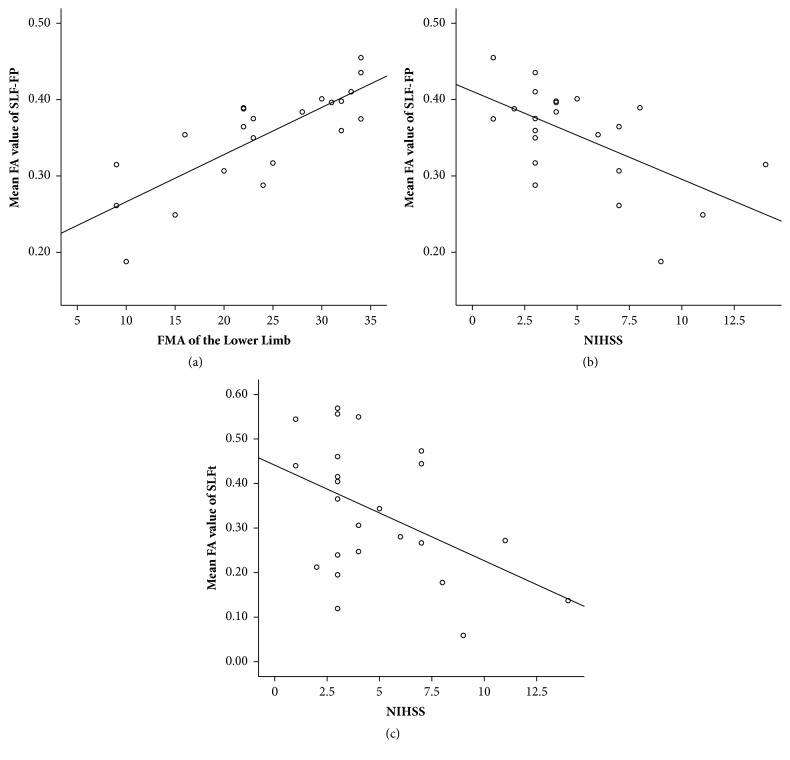
Correlation between FA and clinical scores. (a) FA value of the ipsilesional SLF-FP had a positive correlation with the FMA-L (*P* < 0.000); (b) FA value of the ipsilesional SLF-FP had a negative correlation with the NIHSS (*P* = 0.004); (c) FA value of the ipsilesional SLFt had a negative correlation with NIHSS (*P* = 0.024). FA, Fractional anisotropy; FMA-L, FMA of the lower limb; NIHSS, National Institute of Health Stroke Scale; SLF-FP, frontoparietal part of the superior longitudinal fasciculus; SLFt, temporal part of the superior longitudinal fasciculus.

The FA value in the frontoparietal part of the superior longitudinal fasciculus (SLF-FP) showed a positive correlation with the Fugl-Meyer Assessment of the lower limb (FMA-L) and a negative correlation with the NIHSS score in stroke patients (Figures 2(a) and 2(b)). A negative relationship was observed between the FA value in the temporal part of the superior longitudinal fasciculus (SLFt) and the NIHSS score (Figure 2(c)).

### 3.4. Origins and Terminations of the SLF

**Figure 3:**
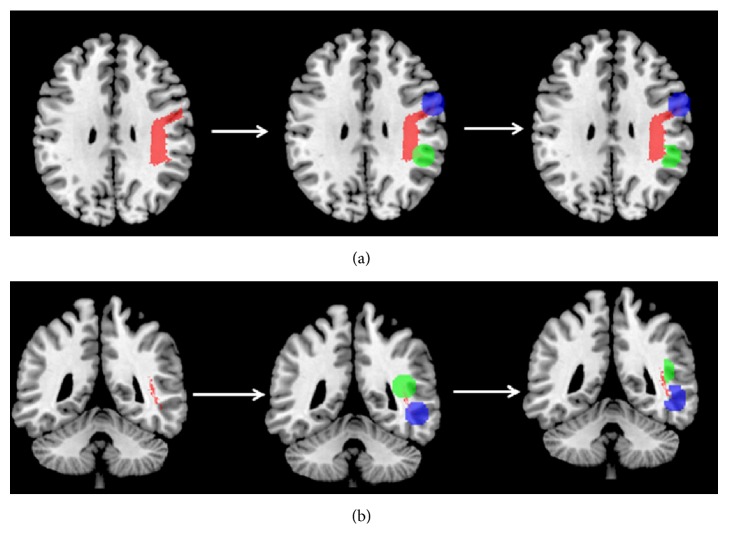
Origins and terminations of the right SLF-FP and SLFt. (a) Brain regions connected by the SLF-FP; (b) brain regions connected by the SLFt. Left panel depicted the course of SLF-FP and SLFt. The middle panel indicated the brain regions, which used the origin or termination of the SLF-FP of SLFt as center coordinates of 10-mm-radius spherical ROIs. The right panel indicated the cortical gray matter of the ROIs. ROI, regions of interest; SLF, superior longitudinal fasciculus; SLF-FP, frontoparietal part of the superior longitudinal fasciculus; SLFt, temporal part of the superior longitudinal fasciculus.

The right SLF-FP originated from the ipsilateral premotor cortex (PM) and the supplementary motor area (SMA) and terminated in the supramarginal gyrus (SMG) (Figure 3(a)). The right SLFt connected the ipsilateral inferior and middle temporal gyri with the primary auditory cortex and the primary association cortex (Figure 3(b)).

### 3.5. Acupuncture Intervention on FC

ANOVA revealed a significant main effect of group (*F*_(1,46)_ = 9.121;* P* = 0.004), but not the main effect of acupuncture condition (*F*_(1,46)_ = 2.166;* P* = 0.149), on FC value of brain regions connecting the right SLF-FP. Moreover, the interaction effect of group and acupuncture condition was significant (*F*_(1,46)_ = 11.407;* P* = 0.002; Table 2).

**Figure 4:**
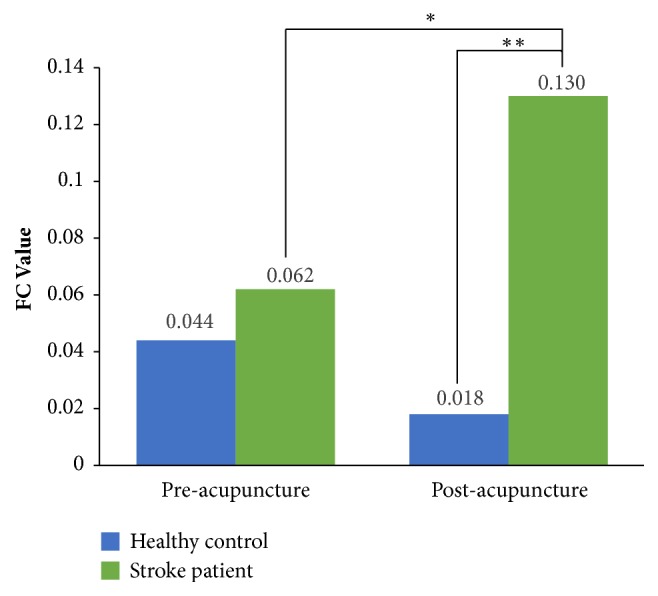
FC alterations before and after acupuncture intervention in stroke patients and healthy controls. Connected by the SLF-FP, the adjoining PM and SMA had conspicuously increased FC with the SMG under acupuncture intervention in stroke patients. The green color indicates the stroke patients. The blue color indicates the healthy controls. *∗P* < 0.01 versus before acupuncture intervention; *∗∗P* < 0.001 versus the group of healthy controls. FC, Functional connectivity; PM, premotor cortex; SLF-FP, frontoparietal part of the superior longitudinal fasciculus; SMA, supplementary motor area; SMG, supramarginal gyrus.

Under acupuncture, the stroke group exhibited an enhanced FC compared with the healthy controls (*P* < 0.001; Table 3 and Figure 4). Nevertheless, no significant differences were found between the two groups during the preacupuncture resting state (*P* = 0.499; Table 3).

**Figure 5:**
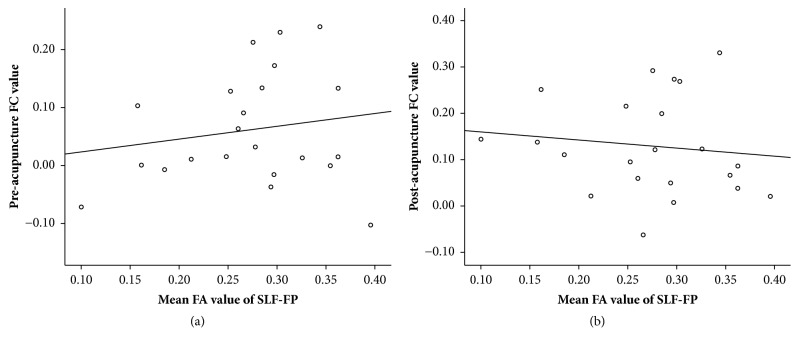
Correlation between the pre- and postacupuncture FC values and the corresponding FA values using Pearson correlation analyses. No significant correlations were observed between the FC value of preacupuncture intervention and the FA value of the corresponding SLF-FP (*P* = 0.448), as well as the FC value of postacupuncture and the corresponding FA value (*P* = 0.588).

Significant connectivity increases (pre and postacupuncture FC changes) were found in the stroke group (*P* = 0.002; Table 4 and Figure 4) but not in the healthy controls (*P* = 0.206; Table 4). In contrast, no significant effect was observed for the FC of brain regions linked by the right SLFt. Also, the main effects or interaction effect was not noted. In addition, the FC values of pre- (*P* = 0.448) and postacupuncture (*P* = 0.588) did not display a correlation with the FA values of corresponding injured white matters (Figure 5).

## 4. Discussion

The motor-related FA changes in stroke patients and the brain FC reorganization under acupuncture intervention were investigated by combining DTI, resting fMRI, and clinical data. The extent of injured microstructure in the right SLF correlated with the motor deficits of the lower limbs following a stroke. The impaired structural integrity of the SLF-FP corresponded well to the connected regions presenting enhanced FC between the PM/SMA and SMG. Acupuncture enhanced FC between these cortical regions in stroke patients, but not in the healthy controls.

A notable disruption in the ipsilesional SLF in stroke patients was found in the present study, which was most likely due to Wallerian degeneration secondary to cerebral infarction. The Wallerian degeneration caused white matter disruptions at sites remote from the initial lesion. It illustrated that the loss of white matter integrity in the SLF was associated with poor motor outcomes of the lower extremity in patients with an infarction on descending motor pathways. However, the extant studies regarding poststroke motor impairment focused on the FA of the CST. The CST is the major white matter motor pathway controlling movements of the limbs and trunk. The CST may be a relevant biomarker of motor function in acute and subacute phases [37, 38]. Shifting into the chronic stage, it is essential to enroll larger and distributed motor networks to explain the deficits and recovery of motor function [10, 27]. Recent studies illustrated that the CST integrity was required for proportional recovery in the upper limbs, but not in the lower limbs [6, 39]. In the present study, the CST also displayed obvious lesions in stroke patients. However, no conspicuous association was observed between the integrity of the CST and the severity of motor impairment. The variability between results might be partly attributable to the wide range of upper limb motor deficits in recruited patients. Moreover, the motion of upper limbs, especially the hands, displayed an individual preference for use, known as dominant hand. In the present study, the participants were all right-handed and the lesions were restricted within the right hemisphere instead of both sides. Thus, the inconsistency between a previous study and the present study might result from the different inclusion criteria and the lateralization of brain function. It is necessary to further investigate the possible discrepancy of the features in stroke patients with different handedness. The findings demonstrated that the SLF, measured using the FA, was related to motor deficits of the lower limb. It implied that the integrity of the SLF might be able to predict motor outcomes of the lower extremity after a subcortical stroke.

The SLF is a large bundle of intrahemispheric association fibers, first depicted by Reil and Autenrieth in 1809. As a corticocortical white matter trajectory, it is the major communication of the temporal, parietal, and frontal regions around the Sylvian fissure [40]. The SLF is considered to be involved in multiple assignments, such as cognitive control [41, 42], motor processing [43, 44], spatial working memory [45, 46], visual–spatial attention [47], and language development [48]. The subcomponents and trajectory of SLF fibers are widely heterogeneous and still disputed [40, 49, 50]. In the present study, two subcomponents of the SLF were identified by correlating the FA value with the clinical assessments, the SLF-FP and SLFt. The SLFt connected the inferior and middle temporal gyri with the auditory primary and association cortex, which was rarely reported earlier. The SLF-FP coursed from the PM and conjoint SMA and inserted into the SMG. It could be identified as part of the SLF-II or dorsal SLF [49].

The premotor cortex (PM) and SMA are frontal brain regions connected by SLF-FP, which are located on Brodmann area 6 and generally regarded as secondary motor areas. As part of the motor circuit, both PM and SMA contributed to the plan and adjustment of gait movements [51, 52]. The SMG, located on the Brodmann area 40, is a portion of the parietal lobe. Gathering evidence manifested that the regions of the parietal lobe, such as the SMG and the intraparietal sulcus, were involved in movement function [52–54]. Nonetheless, the functional interactions among these cortical motor areas are still far from fully understood. Neuroimaging studies have revealed that the corticocortical structural integrity and functional interactions between the primary motor cortex (M1) and secondary motor areas contribute to hand or upper limb motor output in patients with a subcortical stroke [17, 55]. However, not much attention has been placed on lower limb motor disability so far. A more extensive set of brain regions should be taken into consideration for the poststroke motor outcome [27], especially for the lower limb. Furthermore, the mirror neuron system, which includes the PM and SMG, underlies the mechanisms of observational learning [56]. The mirror neuron system is a neural substrate for therapies of stroke [57]. The secondary motor areas and the SMG, which are initially relevant to complicated movement and learning, may take over the deficient motor function following a stroke. Therefore, the integrity of the ipsilesional SLF-FP may play a vital role in poststroke motor deficits.

Acupuncture could modulate interhemispheric and intrahemispheric FC among multiple cortical motor areas, implicating brain plasticity in the motor-related network in a stroke with a unilateral subcortical lesion [58, 59]. For instance, acupuncture intervention can improve declined FC between the bilateral M1 in stroke patients [22]. It is particularly necessary and useful to describe the disturbances in FC following a structural lesion in a stroke by combining structural and functional information [60, 61]. Based on DTI, the impaired SLF subcomponents were figured out according to the correlation with motor scales in the present study. The FC value was calculated between brain regions corresponding to these damaged white matter fibers. The PM and neighboring SMA had significantly increased FC with the SMG under the acupuncture intervention in stroke patients. Conversely, healthy controls showed no marked difference in the FC of the aforementioned regions between pre- and postacupuncture intervention. A previous study, which also applied acupuncture at GB34, observed the activation on similar brain regions. In contrast to sham stimulation, acupuncture could make an impact on the bilateral somatomotor cortices (BA 3, 4, 6, and 7) [62]. The PM, SMA, and primary motor cortex (M1) within the ipsilesional motor network had complicated interactions closely relevant to motor function [52, 63]. The results suggested that the modulation of communication between the regions connected by the ipsilesional SLF-FP could be a potential mechanism underlying poststroke acupuncture intervention. Acupuncture might be able to promote motor relearning and recovery by enhancing and integrating functional activities of more extensive brain regions, which initially were involved in executive function processes such as planning, initiation, and attention. Additionally, FC of the brain areas linked by the SLFt—the injured white matters correlated with the stroke severity—was not altered by the acupuncture intervention using GB34. These regions were related mainly to the auditory and recognition function. The GB34 is a well-known acupoint for motor disability treatment [25, 34]. The findings indicated that acupuncture on GB34 was able to modulate the brain function related to motion but not hearing and recognition. This may further support the opinion of acupoint specificity [64]. In addition, no statistical correlation was found between the impaired FA and the corresponding FC values. It meant that acupuncture intervention could still enhance the cortical communication even under the connection of injured white matter tracts, and the extent of functional alterations by acupuncture intervention was not dependent on the integrity of the structural substrate.

Several mechanisms underlying the beneficial effects of acupuncture for a stroke have been illustrated. The mechanisms include regulation of cerebral blood flow, promotion of neurogenesis and cell proliferation, antiapoptosis in the ischemic area, modulation of neurochemicals, and improvement in impaired long-term potentiation after an ischemic stroke [65]. Based on these facts, it is possible that acupuncture provokes and integrates the motor-related function of the cortices.

Future studies should apply other therapeutic strategies, such as applying transcranial magnetic stimulation, on these brain regions. Additionally, it was interesting that the acupuncture-induced changes in FC just existed in the poststroke situation but not in a healthy state. It seemed to imply the functional specificity of acupuncture, meaning that acupuncture was able only to exert particular effects in an abnormal situation. It also indicated that the acupuncture fMRI studies under pathological condition were crucial.

The present study had some limitations. First, multiple variables, including stroke duration, infarct volume, and cognitive level, were inevitably involved in neuroimaging studies. The unilateral subcortical infarct in the present study was restricted to the right cerebral hemisphere. Additionally, the duration was limited to 3 months after onset. Thereby, future studies with larger sample sizes should take these individual differences into consideration to verify the present findings. Moreover, it is also very necessary to study the therapeutic effect of acupuncture with a group of acupoints and a course of treatment, which is in line with the clinical practice. The results of the current study could lay the foundation for further substantiating the dynamic evolution of structural and functional alterations on long-term acupuncture treatment.

## 5. Conclusions

In conclusion, the results of the present study indicated that the structural damage of the ipsilesional SLF had an important role in poststroke motor deficits of the lower limbs. Hence, the integrity of SLF might serve as a neuroimaging biomarker for the motor outcome in the lower limbs following a stroke. After the acupuncture intervention, a conspicuous enhancement was observed on the FC between the PM/SMA and the SMG, which were the cortical origin and termination of the SLF-FP. The communication of the cortices could still be increased by acupuncture even with the connection of the injured white matter tracts. This implied that the instant neural effect of acupuncture intervention did not depend on structural integrity. The findings might provide an insight into the mechanisms of acupuncture intervention for motor impairment following a stroke.

## Figures and Tables

**Table 1:** Group demographics and relevant clinical features.

Subject	Sex	Age (year)	Stroke duration (day)	NIHSS	FMA-U	FMA-L
Patient	15M/7F	59.91 ± 7.65	41.68 ± 25.02	5.05 ± 3.29	34.27 ± 19.80	24.50 ± 8.62
Control	10M/12F	57.95 ± 5.21	n/a	n/a	n/a	n/a

F, female; FMA-L, Fugl-Meyer Assessment of the lower limb; FMA-U, Fugl-Meyer Assessment of upper limb; M, male; NIHSS, National Institute of Health Stroke Scale.

**Table 2:** ANOVA results on FC alterations on the 2 × 2 factorial design: stroke patients or healthy controls (intersubject) × pre- or postacupuncture (within-subject).

	SLF-FP		SLFt	
	*F*_(1,46)_	*P* value	*F*_(1,46)_	*P* value
Group of participants	9.121	0.004*∗*	2.717	0.107
Acupuncture condition	2.166	0.149	0.451	0.505
Interaction effect	11.407	0.002*∗*	0.549	0.463

The main effect for two groups (stroke patients versus healthy controls) and the interaction effect were significant in the FC between brain regions connected by the SLF-FP. In contrast, the FC of brain regions linked by the right SLFt was not significant for the main effects or the interaction effect.

**Table 3:** Two-sample *t* test to compare the FC values between two groups pre- and postacupuncture.

Acupuncture condition	Group of participants	FC (X ± S)	*T* value	*P* value
Preacupuncture	Stroke patients	0.062 ± 0.096	–0.682	0.499
	Healthy controls	0.044 ± 0.070		
Postacupuncture	Stroke patients	0.130 ± 0.106	–4.355	< 0.001
	Healthy controls	0.018 ± 0.057		

No significant differences were found between the two groups in the preacupuncture resting state. After acupuncture, the stroke group exhibited prominently greater enhanced FC compared with the healthy controls.

**Table 4:** Paired *t* test to compare the FC values between pre- and postacupuncture in stroke patients and healthy controls.

Group of participants	Acupuncture condition	FC (X ± S)	*T* value	*P* value
Stroke patients	Preacupuncture	0.062 ± 0.096	–3.544	0.002
	Postacupuncture	0.130 ± 0.106		
Healthy controls	Preacupuncture	0.044 ± 0.070	1.306	0.206
	Postacupuncture	0.018 ± 0.057		

A significant connectivity increase (pre- and postacupuncture FC changes) was observed in the stroke group but not in the healthy controls.

## Data Availability

The data used to support the findings of this study are available from the corresponding author upon request.

## Supplementary Materials

Supplementary Materials Supplementary Figure 1: experiment design of MRI scan and acupuncture intervention. The paradigm consisted of a resting-state fMRI, a DTI scan, an fMRI scan accompanied by acupuncture intervention, and a T1 structural scan in order. In the third step, the fMRI was acquired at the end of the 1-min manipulation, and the needle was removed after this scanning.
