# Supplementary material for: Acupuncture Enhances Communication between Cortices with Damaged White Matters in Poststroke Motor Impairment
Source: Evid Based Complement Alternat Med. 2019 Jan 2;2019:4245753. doi: 10.1155/2019/4245753 (PMC6334314; doi:10.1155/2019/4245753)
Supplement: Supplementary Materials — Supplementary Figure 1: experiment design of MRI scan and acupuncture intervention. The paradigm consisted of a resting-state fMRI, a DTI scan, an fMRI scan accompanied by acupuncture intervention, and a T1 structural scan in order. In the third step, the fMRI was acquired at the end of the 1-min manipulation, and the needle was removed after this scanning. [file 4245753.f1.pptx]

## Slide 1
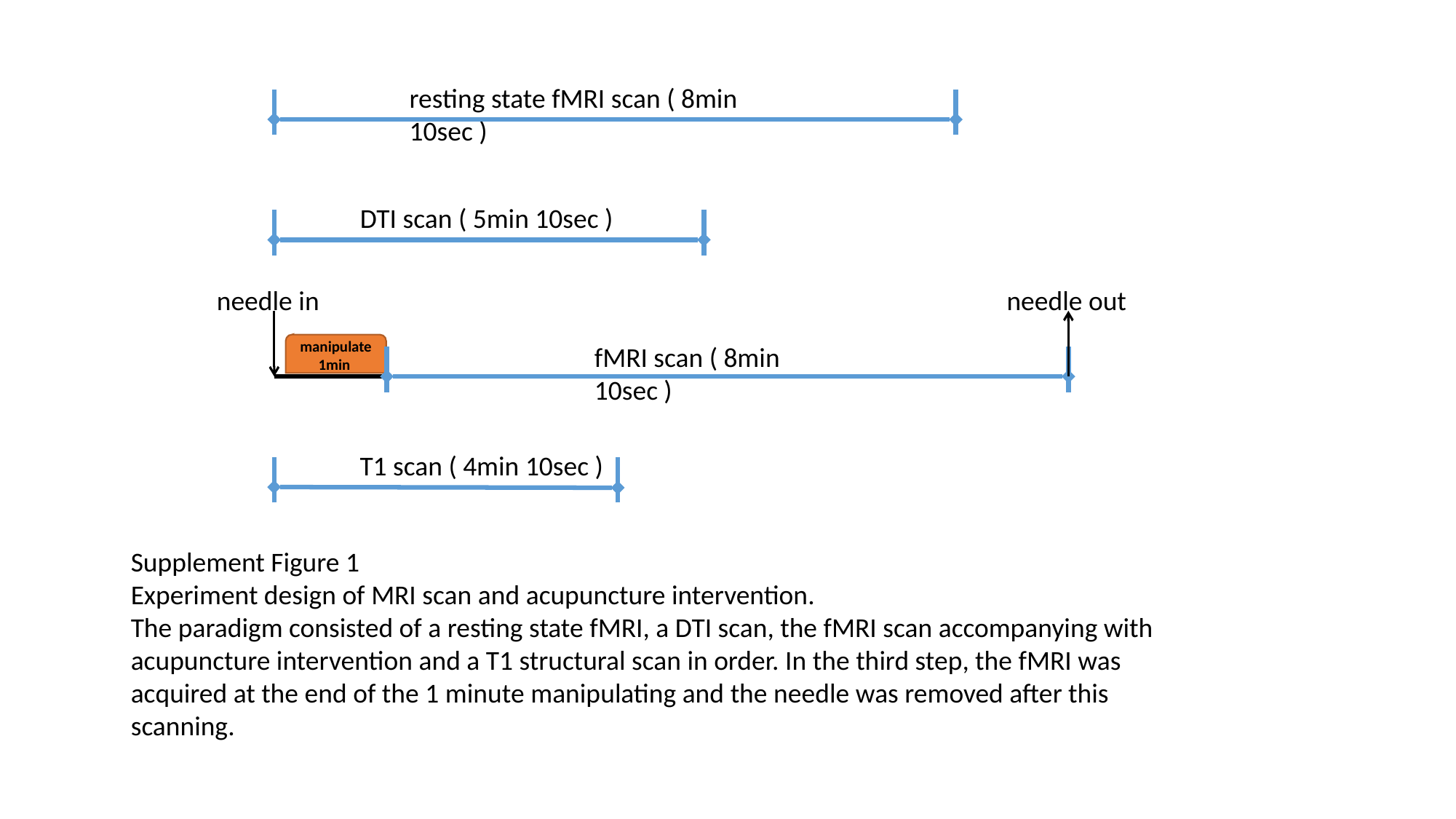

resting state fMRI scan ( 8min 10sec )
DTI scan ( 5min 10sec )
needle in
needle out
manipulate 1min
fMRI scan ( 8min 10sec )
T1 scan ( 4min 10sec )
Supplement Figure 1
Experiment design of MRI scan and acupuncture intervention.
The paradigm consisted of a resting state fMRI, a DTI scan, the fMRI scan accompanying with acupuncture intervention and a T1 structural scan in order. In the third step, the fMRI was acquired at the end of the 1 minute manipulating and the needle was removed after this scanning.
